# Nationwide Study of Turner Syndrome in Ukrainian Children: Prevalence, Genetic Variants and Phenotypic Features

**DOI:** 10.4274/jcrpe.5119

**Published:** 2018-07-31

**Authors:** Nataliya Zelinska, Iryna Shevchenko, Evgenia Globa

**Affiliations:** 1 Ukrainian Research and Practical Center of Endocrine Surgery, Transplantation of Endocrine Organs and Tissues of the Ministry of Health of Ukraine, Department of Pediatric Endocrinology, Kyiv, Ukraine

**Keywords:** Turner syndrome, prevalence, growth retardation, karyotype, phenotype, malformations

## Abstract

**Objective:**

We aimed to investigate the prevalence of Turner syndrome (TS) in the Ukrainian population, the frequency of karyotype variants, the age of children at diagnosis, the degree of short stature and phenotypic features in TS girls.

**Methods:**

A retrospective analysis was made in 538 TS girls aged 0.11-18.2 years within the time period of 2005-2015 with detailed examination of 150 patients.

**Results:**

The prevalence of TS in Ukraine is 77.5 in 100.000 live female births. The average age at diagnosis is 9.33±4.93 years. The relative proportions of karyotypic abnormalities found were: 45,X (59.3%); mosaicism 45,X/46,XX (22.9%); and structural abnormalities in chromosome X (17.8%). The most frequently encountered findings were growth delay (98.8%), shortening of the 4^th^ and 5^th^ metacarpal bones (74.6%), abnormal nails (73.3%), broad chest (60.7%), short neck (58.6%), hypertelorism of nipples (51.4%), malformations of the cardiovascular (19.6%) and urinary systems (13.8%) and pathology related to vision (20.1%) and hearing (22.0%).

**Conclusion:**

In the Ukrainian population, the highest proportion of patients with TS had a karyotype 45,X. TS was accompanied by a lower frequency of malformations of internal organs compared to other countries.

## What is already known on this topic?

Turner syndrome (TS) is one of the most common genetic disorders associated with abnormalities of chromosome X. A significant delay at initial diagnosis of TS has been reported in all populations. 

## What this study adds?

The most common karyotype in Ukrainian Turner syndrome (TS) patients was 45,X. In this population, TS was accompanied by a lower frequency of cardiac and renal malformations compared to other countries.

## Introduction

Turner syndrome (TS) is one of the most common genetic disorders associated with abnormalities of chromosome X (1). The incidence of this syndrome has been reported to vary from 25 to 210 per 100.000 live female births in different populations. This variability has been attributed to the prevalence of mosaic forms and the lack of classical features of the disease (1,2,3,4,5). A significant delay at initial diagnosis of TS has been well documented (3,6,7,8). The average age of TS diagnosis varies widely from 4.1±5.1 years in the United State of America (USA) up to 13.74 years old in Albania (6,9,10,11). Diagnosis in patients with a 45,X karyotype is usually made at an earlier age than other variants (9). Karyotype 45,X has been reported in between 32-74% patients, while 9.2-31% patients are carriers of different variants of chromosome X structural abnormalities and a mosaic karyotype is present in between 9-56.3% of patients (12,13,14,15,16,17,18,19). Delay in growth is commonly associated with TS and is present in most affected girls. Final height in 45,X patients who did not receive treatment with recombinant growth hormone (rGH) was typically less than 142-145 cm, which is about 20 cm lower than the average height of a healthy female population (20,21). The most serious malformations of TS include congenital and acquired heart diseases, such as aneurysm and aortic dissection, valvular disease, hypertension, thromboembolic disease and myocardial infarction (22,23). Sybert and McCauley (23) reported cardiac pathology in 56% of TS patients. Defects of the urinary tract, including pyelocaliceal system defects, horseshoe kidney and other anomalies of kidney location are observed in 30-40% of TS patients (24,25). Epicanthus, palpebral ptosis and strabismus are the major stigmata of disembryogenesis of eyes and eye appendages encountered in patients with TS (26,27). Hearing defects in patients with TS are characterized by a high frequency of otitis media, assumed to be caused by abnormalities in the eustachian tube and middle ear (20,28,29). Patients with TS are reported to have a higher frequency of autoimmune diseases, including autoimmune thyroid disease and celiac disease (30,31,32,33). Several researchers in Europe have reported increased levels of antithyroid antibodies in patients with TS, the frequency ranging from 36 to 64.8% (6,30,31,32,33,34,35). Of these, 21.2-70.4% were reported to have subclinical or clinical hypothyroidism (30,31,33,34,35). The aims of our study were to investigate the prevalence of TS among children in Ukraine. In addition we also aimed to determine age at initial diagnosis, frequency of different karyotype variants, degree of growth delay and prevalence of different phenotypic features of TS.

## Methods

In this study the Ukrainian Pediatric TS Registry, created in 2004, was used. The registry included children diagnosed with TS between ages 0.11 and 18.2 years old, identified by regional Ukrainian pediatric endocrinologists. TS registration cards contained information on date of birth, age at diagnosis, karyotype, height (cm), height standard deviation (SD) [World Health Organization (WHO), 2007] (36), weight (kg), body mass index [BMI (kg/m²)] derived from WHO percentile tables for girls of appropriate age (36) and Tanner stage of sexual development (37). Data on phenotypic features of the girls was also recorded. Biochemical and hormonal parameters available from the records included: thyroid stimulating hormone (TSH), free thyroxine (fT4), thyroid peroxidase antibodies (TPOAb), luteinising hormone, follicle stimulating hormone, estradiol, insulin-like growth factor-1, results of clonidine stimulation test for GH, bone age according to the Greulich and Pyle method. A retrospective analysis of 538 registration cards of TS girls who were registered between the years 2005 and 2015 was conducted. Depending on the karyotype’s variant, the patients were divided into three groups. The first group included patients with 45,X (n=319). The second group consisted of patients with the mosaic karyotype 45,X/46,XX (n=123) and the third group of girls had structural abnormalities of chromosome X, such as 46,Xi(Xq), 45,X/46,Xİ(Xq), 45,X/46,X+mar, 46,X, del(X)(Xq) and 45,X/46,Xdel (n=96). The physical development of girls with TS were compared with healthy girls in the control group aged from 10 months to 18 years old. The control group included 525 healthy girls. This group had been under observation during the health-care examinations in 2005-2008. The exams took place in our Clinic and in preschools and schools of the Ukraine. All girls in the study and control groups were divided into 5 age groups as follows: younger than 1 year of age; 1 to 3 years; 4 to 7 years; 8 to 11 years and older than 12 years of age. In addition 150 girls with TS were examined in the Ukrainian Research Center of Endocrine Surgery of the Ministry of Health of Ukraine and in the National Children’s Specialized Hospital “OHMATDYT”. Diagnosis of TS was confirmed by determination of karyotype in blood leukocytes. In these 150 all parameters from TS registration cards were re-evaluated. To study anomalies of internal organs, these patients underwent ultrasound of internal organs and echocardiography. An audiogram was performed in all patients with hearing loss. Also, all children were examined by an ophthalmologist and otolaryngologist to confirm the presence or absence of any abnormal features. To assess thyroid function in patients with different TS karyotype, thyroid function tests (TSH and fT4) and TPOAb concentrations were determined. In the scope of our study, we did not assess the prevalence of celiac disease.

### Statistical Analysis

Statistical analysis of the results was performed by using Statistica 10 (StatSoft, USA). Standard non-parametric statistical tests and Kruskal-Wallis test or Student’s test in the case of normal distribution were used. For the analysis of qualitative data (%) for two or more independent groups χ^2 ^Pearson was used. One-Way analysis of variance test was used for the quantitative data analyses in groups. The data are presented as mean values ± SD or as median and 25^th^ and 75^th^ percentiles (first and third quartiles) [median (25; 75)] for parametric and nonparametric distributions respectively. A p value of <0.05 was taken as an indicator of significant difference. This study was approved by the Ethics Committee of Ukrainian Research Center of Endocrine Surgery MoH of Ukraine (approval number: 12 from 14.10.2013). All procedures performed in the studies involving patients were in accordance with the ethical standards of the Institution on clinical practice and with the 1964 Helsinki Declaration, as amended. The parents or legal guardians of patients signed informed consent forms in which they agreed to the treatment and all the diagnostic procedures required.

## Results

Data from the Ukrainian Pediatric TS Registry shows that the prevalence of TS among children aged 0-18 years was 77.5 per 100.000 female live births during the study period. Over the last five years there were 17-25 new TS cases with registered annually (38). Among girls with TS (n=538), different karyotype variants were found. However, monosomy 45,X was identified most often, in 59.32% of the patients (p<0.001), than mosaicism 45,X/46,XX (22.90%) and structural abnormalities of chromosome X in 17.78% patients. The structural abnormalities were further divided as follows: 46,Xi(Xq) in 5.11%; 45,X/46,XX(Xq) in 6.9%; 45,X/46,X+mar in 3.16%; 46,X,del(X)(Xq) in 1.87%; and 45,X/46,X,del in 0.74% of patients. In Ukraine the mean age of diagnosis of TS in children was 9.33±4.93 years. However, age of diagnosis depended on the karyotype and was lowest in children with 45,X as compared to children with structural abnormalities of chromosome X (p=0.013), Age of diagnosis was 8.96±5.28 years in 45,X patients (n=303), 10.49±3.95 years in patients with structural abnormalities of chromosome X (n=87) and 9.50±4.41 years in 45,X/46,XX (n=111) patients. TS was diagnosed in the first year of life in 1.62%, at ages 1-4 years in 3.60%, at ages 5-7 years in 9.46%, at 8-11 years (the age when normal puberty is expected to start in girls) in 18.92% and between 12-17 years, when puberty would be expected to have begun in 66.40% of the patients. Phenotypic manifestations of TS in children had significant variability. Growth delay was a constant feature (98.82% of patients). Shortening of 4^th^ and 5^th^ metacarpal bones (74.62% patients) followed by abnormal nails (73.31%), broad chest (60.67%), short neck (58.63%), sexual developmental delay (57.32%) and hypertelorism of nipples (51.37%) were the most frequently observed findings. These manifestations of TS were most frequent in patients with karyotype 45,X and significantly less frequent in patients with 45,X/46,XX (p=0.023) and structural abnormalities of chromosome X (p=0.035).

Malformations of the cardiovascular system was the most common pathology of internal organs in TS patients (19.62%). These consisted of aortic stenosis (in 5.32% of the patients), coarctation of the aorta and bicuspid aortic valve (in 2.63% and 2.02% of the cases respectively). Malformations of the cardiovascular system were found more often in children with mosaicism (26.18%) and in cases of structural abnormalities of chromosome X (21.62%) compared to the karyotype 45,X (15.85%) (p=0.02). Urinary tract malformations were observed less frequently in the patients (13.82%) but were significantly more common (p=0.017) in patients with karyotype 45,X (14.76%) and less so in cases with karyotype 45,X/46,XX (8.28%) and structural abnormalities of chromosome X (2.75%). The main malformations of urinary tract were doubling pyelocaliceal renal system (3.38%), renal hypoplasia (3.36%) and ureter malformations (3.31%). Frequency of vision and hearing defects were 20.08% and 22.01% respectively. Otitis was the most common pathology pertaining to the ears and was observed most frequently in children with monosomy X (p<0.01). Optic and otic pathologies found are shown in Table 1. Frequency of autoimmune thyroid disease in TS girls was 48.45%. It was proved by elevated levels of TPOAb and appropriate ultrasound changes, and it did not differ significantly between karyotypes (p>0.05). Among TS girls with elevated TPOAb levels subclinical (48.76%) and clinical hypothyroidism (29.14%, p<0.05) were found more frequently. 11.87% of patients were euthyroid, and 10.23% of girls had subclinical hyperthyroidism. Until 2013, in Ukraine there was no state programme of free treatment with rGH for girls with TS, thus most patients were untreated. The analysis of growth in girls with TS who did not receive treatment with rGH compared to the control group of appropriate age revealed a significant difference in the growth of children in all age groups (Table 2). The difference was noticeable in the first year of life, increased with age and was highest in those girls aged 14 years old, probably due to the lack of pubertal growth spurt in TS patients. The difference in final height between TS girls and controls was 24.4±1.7 cm (p<0.001). Analysis of final height in girls who did not receive rGH revealed no significant difference among patients with different karyotypes (Table 3). In our TS group of pubertal age, spontaneous (without hormonal stimulation) sexual development, assessed by the appearance of thelarche, occurred in 14.62% (n=18). There was no significant difference in the age of onset of puberty among groups with different karyotypes (Table 4). 

Spontaneous menstruations was reported in 38.82% of TS girls having signs of sexual development. It is of note that among girls with spontaneous puberty and karyotype 45,X, there were three girls at Tanner stage 2, three at Tanner stage 3 after the age of 15 years and three girls who had spontaneous menarche at age of 15.1 (14.30; 16.20) years. The frequency of body weight disorders in TS girls was assessed by comparing their BMI values with that of girls of similar age in the control group. Most of the TS girls (68.1%) had a normal BMI (p=0.021), although 13.82% were overweight, 6.88% were obese and 11.20% were underweight (BMI ˂15^th^ centile). The average BMI in patients with TS was 53.23±27.06^th^ percentile that was significantly higher (p<0.05) compared to the control group (50.62±27.63^th^ percentile) (Table 5). According to our data, BMI in patients with TS increased with age and reached a maximum in children over the age of 12 years, but did not exceed the normal range. The highest BMI values were found in girls with structural abnormalities of chromosome X (p<0.05) (Table 6).

## Discussion

The current study aimed to assess the prevalence, age at initial diagnosis, incidence of different variants of the karyotype, phenotypic characteristics, presence of associated components and physical and sexual development in Ukrainian TS patients. It was found that in 2015 the prevalence of TS among children 0-18 years in Ukraine was 77.5 per 100.000 live female births. The incidence rate is consistent with other reports, although is a higher figure when compared to some other countries such as Denmark (3,5), Germany (9), Albania (6) and Japan (2). Age of initial diagnosis of TS was 9.33±4.93 years with a maximum frequency of initial registration of the disease in puberty, most likely because of the referrals of patients with growth delay or delay/absence of sexual development or menstruation at normal female pubertal ages. The highest proportion of early primary diagnosis of TS in Ukraine was found in patients with karyotype 45,X, who were diagnosed at a mean age of 8.96±5.28 years, a finding which can be explained by the presence of typical features of the disease in these girls. Though the age of the diagnosis is older than in a Belgian population (where the average age of diagnosis in 2003 was 6.6 years (9)), it was lower than in a Denmark (where the average age at diagnosis for the entire TS group was reported as 15.1 years, or 13.3 years for 45, X patients) (11). In Ukraine, the largest proportion of patients diagnosed with TS (59.32%) were 45,X, similar to patients from Poland, United Kingdom (UK) and USA (17,18,19). Diagnosis of different karyotype may vary depending on the different methods of analysis and the type of biological material that has been used. In our study, only cytogenetic analysis of peripheral blood lymphocytes was used to determine the karyotype. However, Hook and Warburton (39) suggested that all live birth girls with karyotype 45,X, actually have a mosaic karyotype because some of their organs and tissues will contain more cell lines with normal or aberrant sex chromosomes, which cannot be determined by peripheral blood sample analysis alone. This may be a rationale for further research of karyotype in other tissues in 45,X girls, especially in those who have signs of spontaneous puberty or mild growth delay. We found a lower frequency of cardiovascular and urinary tract malformations in TS girls compared to the USA (40,41,42), UK (43), Egypt (44), Denmark (45) and France (46). The lower frequency of malformations of the cardiovascular system may be explained by the lack of routine cardiac magnetic resonance imaging (MRI) in girls with TS, in the absence of clinical symptoms in our country. Thus we believe that these results are in need of further confirmation by focused examination to detect the above mentioned pathology. Even in the absence of clinical manifestations, presence of aortic dilatation and associated abnormalities need to be evaluated in pediatric patients with TS. The frequency of pathology of vision and hearing was also lower in Ukraine compared to other countries (20,27,28,29). The lower frequency of hearing defects can be due to the fact that only patients with complaints on hearing loss were assesed by audiogram. TS girls in the Ukraine appear to have a lower frequency of malformations of internal organs. However, we believe that the insufficient diagnostics of both the cardiovascular system and that of auricular pathology need to be taken into account in this conclusion.

Studies on the frequency of autoimmune thyroid disease have shown increased TPOAb levels in 48.45% of our patients, a finding that is similar to figures reported from Italy (32) and Denmark (33), but which is less than in Albania (6) and Poland (35). Also, among TS girls with elevated TPOAb levels the number of patients with subclinical and clinical hypothyroidism was greater (77.9%) as compared to other European countries. Thus, researchers reported 31.4% of patients with subclinical hypothyroidism in Poland, Silesia (30), 21.2% with clinical and subclinical hypothyroidism in Italy (31), 33% with clinical hypothyroidism in Denmark (33) and 24% with hypothyroidism among all patients with TS and 65% patients with positive thyroid antibodies in Greece (34); other authors from Poland (Warsaw) reported 20% of patients with subclinical hypothyroidism (35). 

Height of girls with TS who did not receive treatment with rGH shows that they were significantly shorter in all age groups, compared with the control group, a finding consistent with other studies (21,22). The progression of the degree of growth delay increased with age and was more pronounced in puberty. Final height in TS patients who did not receive treatment with rGH was significantly lower, compared to healthy Ukrainian women.

Most girls with TS (68.1%) had normal body weight. However, overweight was detected in 13.82% of patients, obesity in 6.88% and 11.20% of the patients were underweight. The frequency of overweight in children with TS was higher than in the general population in all age groups with a significant difference in puberty, which coincides with the findings of other authors (47,48,49). The highest BMI and the highest rate of overweight were observed in patients with structural abnormalities of chromosome X. To our knowledge, this data had not been described previously. It is well known that TS is a syndrome of disproportionate anthropometry and body composition. Dual-energy X-ray absorptiometry (DEXA) can be helpful to estimate the visceral fat and skeletal muscle mass. We can hypothesize that TS girls with structural abnormalities of chromosome X (especially those with isochromosome Xq) might have skewed body composition with increased skeletal muscle mass versus total fat mass and hence, increased BMI. In Ukrainian population among structural abnormalities of chromosome X majority patients were girls with isochromosome Xq abnormalities. In our study we used BMI only, that may not reflect the real body composition, however only few authors investigated the effect of karyotype on body composition in children (12,50). Further studies (including those with using of DEXA) are needed to estimate the total and regional distribution of fat and muscle mass in girls with different karyotypes.

### Study Limitations

There are several limitations to this study. There was no national screening for celiac disease. Also we did not perform the evaluation of the hearing loss and MRI of the heart for all TS patients. DEXA with evaluation of the total and regional distribution of fat and muscle mass in girls with different karyotype is needed for the appropriate BMI evaluation.

## Conclusion

The highest proportion of patients with TS in Ukraine had a karyotype 45,X. TS was accompanied by a lower frequency of malformations of internal organs as compared to figures from other countries. This leads us to conclude that more widespread use of cardiac MRI, audiogram screening and celiac disease in all patients with TS in Ukraine is urgently needed. Additionally, the implementation of genetic testing to identify genes associated with malformations (*ZFYVE9, TIMP1, PRKX, KDM6A*) can lead to a higher detection rate of aortic aneurysm formation, congenital urinary malformations and other anomalies (51). Earlier diagnosis of TS would allow more timely medical, psychological and social assistance for girls with TS and their families. It is assumed that pediatricians and family physicians will provide an active and targeted search for TS among girls, especially in those with a delay of growth and sexual development. This targeted approach would contribute to the prevention of short stature by earlier rGH therapy. Expanded screening for malformations, especially cardiac and renal malformation which TS girls are particularly at risk of would also serve to ameliorate some of the associated morbidity seen in TS patients.

## Figures and Tables

**Table 1:**
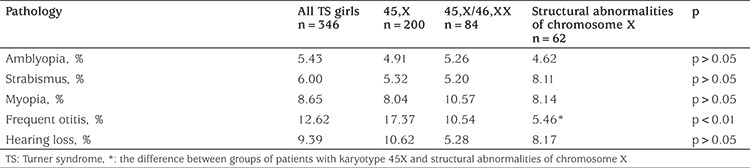
The spectrum of pathology of vision and hearing in Turner syndrome girls with different karyotypes

**Table 2:**
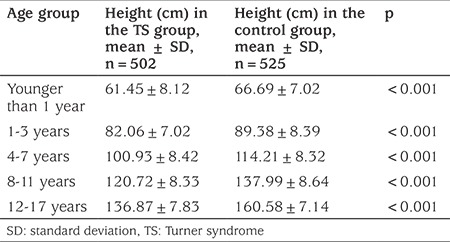
Height in Turner syndrome girls (not receiving recombinant growth hormone treatment) and in the control group, in different age groups

**Table 3:**
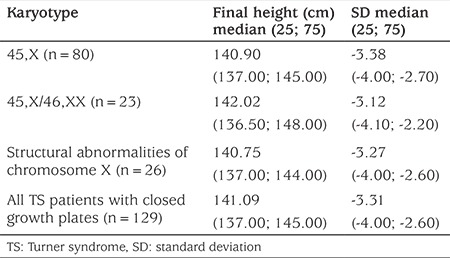
Final height in Turner syndrome girls with different karyotype

**Table 4:**
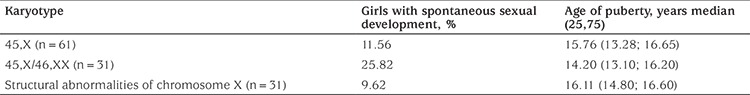
Frequency of spontaneous sexual development and age of puberty in Turner syndrome girls with different karyotypes

**Table 5:**
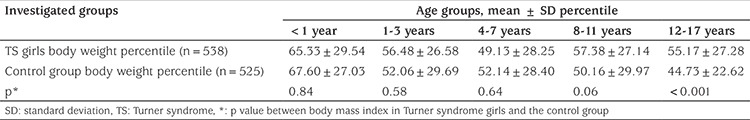
Assessment of body weight using percentile tables in children of all ages with Turner syndrome

**Table 6:**
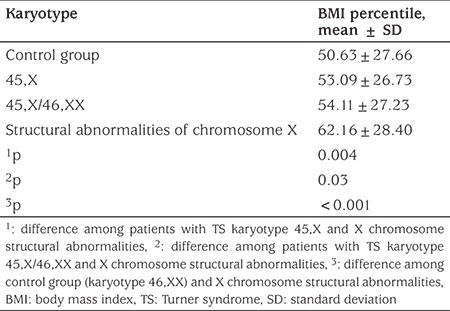
Body mass index percentile in children with Turner syndrome of different karyotypes
